# Microbial Profiles in Oral Lichen Planus: Comparisons with Healthy Controls and Erosive vs. Non-Erosive Subtypes

**DOI:** 10.3390/diagnostics14080828

**Published:** 2024-04-17

**Authors:** Hye-Min Ju, Yong-Woo Ahn, Soo-Min Ok, Sung-Hee Jeong, Hee-Sam Na, Jin Chung

**Affiliations:** 1 Department of Oral Medicine, Dental and Life Science Institute, School of Dentistry, Pusan National University, Busandaehak-ro 49, Mulgeum-eup, Yangsan 50612, Republic of Korea; jc2wma@pusan.ac.kr (H.-M.J.); ahnyongw@pusan.ac.kr (Y.-W.A.); oksoomin@pusan.ac.kr (S.-M.O.); drcookie@pusan.ac.kr (S.-H.J.); 2 Department of Oral Medicine, Dental Research Institute, School of Dentistry, Pusan National University, Busandaehak-ro 49, Mulgeum-eup, Yangsan 50612, Republic of Korea; 3 Department of Oral Microbiology, School of Dentistry, Pusan National University, Yangsan 50612, Republic of Korea; 4 Oral Genomics Research Center, Pusan National University, Yangsan 50612, Republic of Korea; 5 Dental Research Institute, BK21 PLUS Project, School of Dentistry, Pusan National University, Yangsan 50612, Republic of Korea

**Keywords:** saliva, oral bacteria, microbiome, oral lichen planus

## Abstract

Recent studies have begun exploring the potential involvement of microbiota in the pathogenesis of oral lichen planus (OLP), yet comprehensive investigations remain limited. Hence, this study aimed to compare the microbial profiles in saliva samples obtained from patients with OLP against those from healthy controls (HC), along with a comparison between erosive (E) and non-erosive (NE) OLP patients. Saliva samples were collected from 60 OLP patients (E: *n* = 25, NE: *n* = 35) and 30 HC individuals. Analysis revealed no significant differences in alpha diversity, as assessed by the Chao1 and Shannon index, across the three groups. However, Bray–Curtis distance analysis indicated a significant disparity in microbiome composition distribution between HC and E-OLP, as well as HC and NE-OLP groups. The six most abundant phyla observed across the groups were Firmicutes, Bacteroidetes, Proteobacteria, Actinobacteria, Fusobacteria, and Saccharibacteria (TM7). Notably, OLP groups exhibited a higher prevalence of Bacteroidetes. *Prevotella* emerged as the predominant genus in the OLP groups, while *Capnocytophaga* showed a relatively higher prevalence in E-OLP compared to NE-OLP. This study’s findings indicate a notable difference in microbiota composition between HC and patients with OLP. Additionally, differences in the microbiome were identified between the E-OLP and NE-OLP groups. The increase in the proportion of certain bacterial species in the oral microbiome suggests that they may exacerbate the inflammatory response and act as antigens for OLP.

## 1. Introduction

In recent years, technological advancements such as next-generation sequencing (NGS) have facilitated numerous studies examining the relationship between systemic diseases and the microbiome [1]. The oral cavity and gut are significant microbial habitats and have a crucial role in diseases related to the microbiome [2]. The oral cavity has the second-highest biomass after the gut. According to the Human Oral Microbiome Database (HOMD), there are approximately 770 bacterial species reported, of which only 58% have been officially named. It is still uncharted territory with a wide variety of species that are unnamed but cultivated and known only as uncultivated phylotypes (eHOMD version 3.1; www.homd.org). The oral cavity contains various microbial habitats, including the buccal mucosa, subgingival plaque, supragingival plaque, palate, saliva, tonsils, tongue, and throat [3]. Saliva is a non-invasive and easily obtained diagnostic sample that can influence other microbiomes, particularly the gut microbiome [3]. Therefore, it is natural to use saliva samples to study the oral microbiome. However, the lack of standardized saliva collection methods has hindered the use of saliva in microbiome research. Lim, Yenkai, et al. [4] found that saliva sample collection methods do not significantly influence salivary microbiome profiles. The human oral microbiome plays a crucial role in protecting the body from external pathogens through proper interactions [5]. However, disruption of the oral microbiome can lead to various oral diseases, including periodontitis, dental caries, and oral mucosal diseases, as well as non-oral diseases such as rheumatoid arthritis, cancer, and diabetes [6,7,8]. Most of the association between the oral microbiome and oral disease is focused on periodontitis. Systemic autoimmune diseases such as RA, systemic lupus erythematosus (SLE), and primary Sjogren’s syndrome (SS) have been studied. Among these, RA patients had higher disease activity with higher serum antibodies to *Porphyromonas gingivalis*, an oral anaerobic bacterium involved in the pathogenesis of periodontitis [9]. Patients with SLE have increased bacteria in the periodontal area compared to controls [10] and periodontal disease has also been associated with SLE, increasing the risk or severity of SLE [11]. Reduced salivation is the most important factor in altering the oral microbiome, but the role of the oral microbiome in the pathogenesis of SS remains unclear [8]. Among oral autoimmune diseases, there are a few studies on oral lichen planus (OLP).

OLP represents a chronic inflammatory disorder affecting the oral mucosa, typically observed in women over the age of 50 [12]. It is T cell-mediated, with the pathogenesis characterized by an exaggerated immune response of T cells to specific antigens. Nevertheless, the precise antigens triggering OLP development remain elusive. Although various factors such as systemic medications, viral infections, and dental restoration materials have been proposed as contributors, their exact roles in OLP development remain incompletely understood [13,14]. Active research persists in identifying diverse antigens and elucidating the connection between the oral microbiome and OLP [15].

There are several types of OLP, including reticular, plaque, atrophic, papular, erosive, and bullous [12]. The two primary types of OLP are non-erosive (NE) and erosive (E) OLP. Asymptomatic patients with typical NE-OLP are often left untreated and only require follow-up checks. However, E-OLP must be treated, as it can cause pain and bleeding, making it difficult for patients to maintain good oral hygiene and leading to secondary infections [16]. Wang, H. et al. [17] reported a higher presence of Th17 cells in E-OLP compared to NE-OLP, while Shiva et al. [18] found an increase in p53 expression, which can serve as a biomarker for cancer, in E-OLP. Although there are studies on the clinical, histological, and immune response differences between E-OLP and NE-OLP, there is a lack of research on the specific differences that lead to the development of NE versus E forms of OLP [16,19,20,21].

Therefore, the study of OLP and the host’s oral microbiome profile using NGS may provide new insights into the pathogenesis of OLP. However, a very limited number of studies have been conducted on various samples, including buccal mucosa swabs, subgingival plaque, and unstimulated saliva samples with small sample sizes, resulting in inconsistent results [15]. Research on the relationship between the oral microbiome and OLP is still in its early stages. Hence, the aim of this study is to compare the microbial profiles between OLP patients and healthy controls (HC), as well as between NE-OLP and E-OLP patients, using unstimulated saliva samples.

## 2. Materials and Methods

### 2.1. Study Population and Sample Collection

Oral samples were collected from all participants at the Department of Oral Medicine, Pusan National University Dental Hospital, Yangsan, Republic of Korea. Participants were instructed to abstain from eating and performing oral hygiene (such as brushing and flossing their teeth) for two hours prior to sampling. They were then asked to rinse their mouths with water and wait at least ten minutes before providing a sample. Saliva was collected by passive drooling into a 2 mL polypropylene tube for a duration of three minutes. The saliva samples from patients with OLP and healthy volunteers were stored at −80 °C for preservation in the Biobank of Pusan National University Dental Hospital. The samples used in this study were provided by the Biobank of Pusan National University Dental Hospital, a member of the Korea Biobank Network (KBN4_A04). This study included a total of 60 patients with OLP and 30 HC subjects. Depending on the clinical presentation, patients with OLP were divided into two groups: E-OLP (*n* = 25) and NE-OLP (*n* = 35). This study examined patients with OLP for the presence of gingival lesions and assessed the severity of OLP using the REU score [22].

### 2.2. Extraction of Genomic DNA and Next-Generation Sequencing

Total DNA was extracted from the buccal and supragingival plaque using a gram-positive DNA purification kit (Lucigen, Biosearch Technology, Novato, CA, USA) following the manufacturer’s instructions. Each sequenced sample was prepared according to the Illumina 16S Metagenomic Sequencing Library protocols to amplify the V1 and V2 region (27F-338R). The barcoded fusion primer sequences used for amplifications were as follows: 27F: 5′- AGA GTT TGA TYM TGG CTC AG -3′ and 338R: 5′- TGC TGC CTC CCG TAG RAG T -3′ [23]. The DNA quality was measured by PicoGreen and NanoDrop ND-1000 spectrophotometer (Thermo Fisher Scientific, Waltham, MA, USA) and stored at −80 °C until use. The purified amplicons were combined in equimolar amounts and subjected to paired-end sequencing using NovaSeq (Illumina, San Diago, CA, USA).

### 2.3. Bioinformatic Analysis, Statistical Analysis, and Visualization

Data were analyzed using SPSS software (version 23.0; SPSS Inc., Chicago, IL, USA). Statistical significance was set at *p* < 0.05. The Shapiro–Wilk test was used to assess whether the data were normally distributed. The chi-square test was used to analyze the sex distribution. Age, salivary flow rate, No. of teeth and NRS between groups were compared by ANOVA and t-test after the normality of data was confirmed by the Shapiro-Wilk test. Basic microbiome analyses have been performed using the QIIME2 (version 2020.6) [16] and associated plugins. To measure alpha diversities, the Choa1 index and Shannon’s index methods were used. Principal coordinate analysis (PCoA) of the Bray–Curtis distance was performed to determine the community structure using the vegan package v2.3-0 in R software v3.2.1. The Kruskal–Wallis test and the non-parametric permutation multivariate analysis of variance (PERMANOVA) tests were used to assess the statistical significance for alpha and beta diversities, respectively. The species of each OTU was determined by a pre-trained Naive Bayes classifier, using the Human Oral Microbiome Database (eHOMD) 16S rRNA Extended RefSeq sequences (version 15.1) [24]. To test the differential abundance of bacterial species between E-OLP, NE-OLP, and HC, linear discriminant analysis effect size (LEfSe) [25] was applied with default settings. 

### 2.4. Data Availability Statement

The raw sequencing data have been deposited in NCBI GenBank under BioProject ID PRJEB73312.

### 2.5. Ethics Statement

All subjects provided their written consent to participate in the study, and the Institutional Review Board of Pusan National University Dental Hospital (2023-05-021-001) approved the protocol. This study was conducted in accordance with the principles of the Declaration of Helsinki for Human Studies.

## 3. Results

### 3.1. Patient Characterization

The demographic and clinical characteristics of the participants are summarized in Table 1. All three groups had a mean age of over 55 years, and there were no significant differences in age or sex among the groups (*p* > 0.05). The numeric rating scale (NRS), representing the subjective pain scale of the patients, did not differ significantly between the E-OLP and NE-OLP groups, although the E-OLP score was slightly higher. All subjects had more than 20 residual teeth, and there were no differences in the number of teeth among the groups (*p* > 0.05). All patients in the E-OLP group presented erosive lesions on the buccal mucosa, but none presented erosive lesions exclusively on the gingiva. Gingival erosive lesions were observed in combination with erosive lesions in other areas. NE-OLP was scored based on the presence of reticular or plaque forms in the gingiva or other sites. Among the study participants, those with a history of smoking included one case of E-OLP and two cases of NE-OLP, with none reporting drinking alcohol. Those with diabetes included one case of HC, three cases of E-OLP, and two cases of NE-OLP, respectively. All participants with diabetes were taking medication and had well-controlled blood sugar levels.

### 3.2. Diversity and Abundance of Microbiome HC, E-OLP, and NE-OLP Group

**Figure 1:**
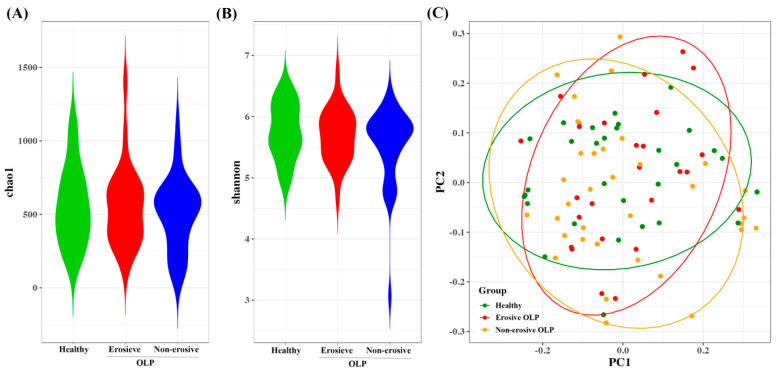
Bacterial community comparisons among healthy, erosive OLP, and non-erosive OLP samples. Alpha diversities (Chao1 (**A**) and Shannon (**B**) indices) of salivary sample. (**C**) Beta diversities of salivary samples. The non-parametric permutation multivariate analysis of variance test was used to identify intergroup differences among the three groups.

The alpha diversity of the microbiota was estimated using the Chao1 and Shannon indices. No significant differences in alpha diversities were observed among HC, E-OLP, and NE-OLP based on these indices (Figure 1A,B). The distribution of microbiome composition was analyzed using the Bray–Curtis distance. The results showed a significant difference between the HC and E-OLP groups, as well as between the HC and NE-OLP groups (*p* < 0.05, as shown in Figure 1C). However, there was no significant difference between the E-OLP and NE-OLP groups (Figure 1C).

**Figure 2:**
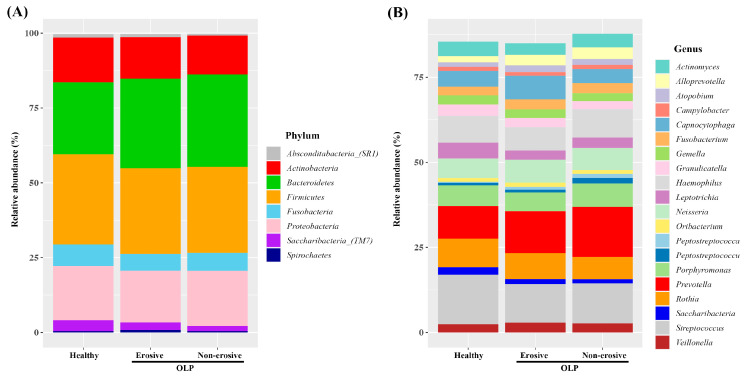
Relative abundances of bacterial species in salivary samples of healthy, erosive OLP, and non-erosive OLP groups. (**A**) At the phylum level and (**B**) at the genus level.

The average relative abundance was assessed at different taxonomic levels. The saliva samples from three groups contained six phyla in the highest abundance: Firmicutes, Bacteroidetes, Proteobacteria, Actinobacteria, Fusobacteria, and Saccharibacteria (TM7). This phylum represents over 97% of the total phylum level. All three groups have a similar bacterial abundance, but Bacteroidetes was relatively more abundant in OLP groups (HC, E-OLP, NE-OLP; 24.0%, 29.9%, 30.9%). In contrast, Saccharibacteria (TM7) was relatively more abundant in the HC group (3.6%, 2.5%, 1.7%) (Figure 2A). A total of 223 genera were detected at the genus level. The most abundant genera in three groups were *Prevotella, Streptococcus, Haemophilus, Rothia, Porphyromonas, Neisseria, Capnocytophaga*, and *Leptotrichia*, representing over 60% of the total sequences (Figure 2B). There was some difference in bacterial abundance between the HC and OLP groups. *Prevotella* was more abundant in the OLP groups than HC (9.6%, 12.3%, 14.7%) and was more abundant than *Streptococcus* in the OLP groups. *Capnocytophaga* was more abundant in E-OLP (6.9%) than in the other two groups, HC (4.7%) and NEOLP (4.1%) (Figure 2B).

### 3.3. Species Taxa Comparison

**Figure 3:**
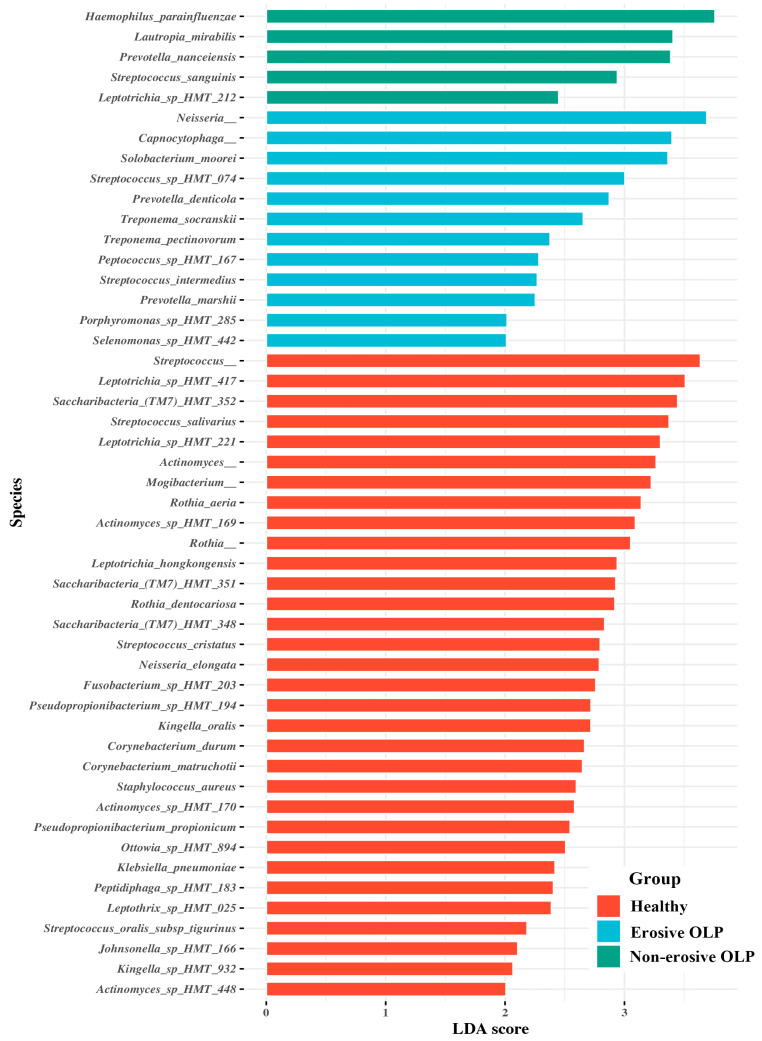
Comparisons of the microbiota abundance in healthy, erosive OLP, and non-erosive OLP samples showing significant differences. This analysis was performed using linear discriminant analysis (LDA) and effect size analysis (LEfSe). Healthy, erosive OLP, and non-erosive OLP-associated bacterial species with a LDA score > 2.5.

**Figure 4:**
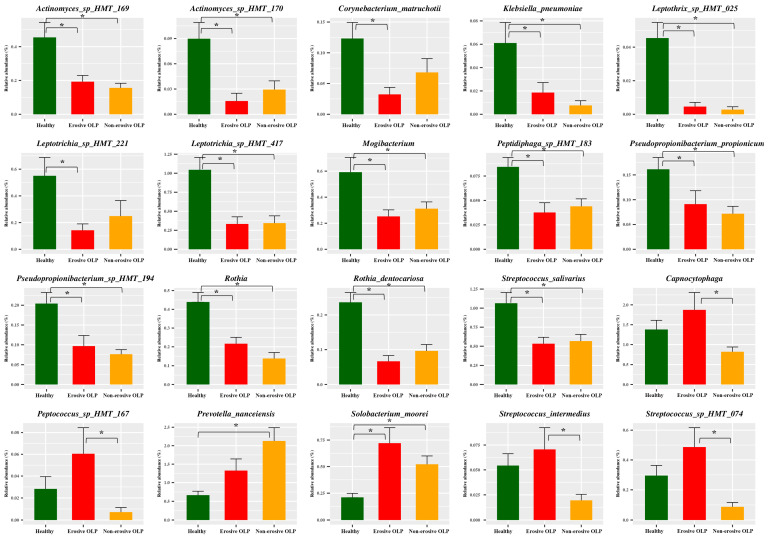
Relative abundance of selected significant bacterial species. The significances of intergroup differences were determined using Tukey’s post hoc test. * *p* < 0.05.

LEfSe was used to evaluate differences in bacterial species abundance among the three groups. Several species were identified as significantly different among the three groups by LEfSe (Figure 3). E-OLPs had higher relative abundances of *Neisseria*, *Capnocytophaga*, *Solobacterium moorei*, *Streptococcus* sp. *HMT 074*, *Prevotella denticola*, and *Treponema socranskii*, while NE-OLPs had higher relative abundances of *Haemophilus parainfluenzae, Lautropia mirabilis, Prevotella nanceiensis*, and *Streptococcus sanguinis* (Figure 3). When Tukey’s post hoc test was applied to the overlapping species, it was found that the abundance of *Capnocytophaga, Peptococcus* sp. *HMT 167*, *Strepococcus intermedius*, and *Streptococcus* sp. *HMT 074* was significantly higher in E-OLP than in NE-OLP. Additionally, *Prevotella nanceiensis* and *Solobacterium moorei* were found to be considerably more abundant in OLP groups than in HC (Figure 4).

### 3.4. Bacterial Interaction Network Analysis

**Figure 5:**
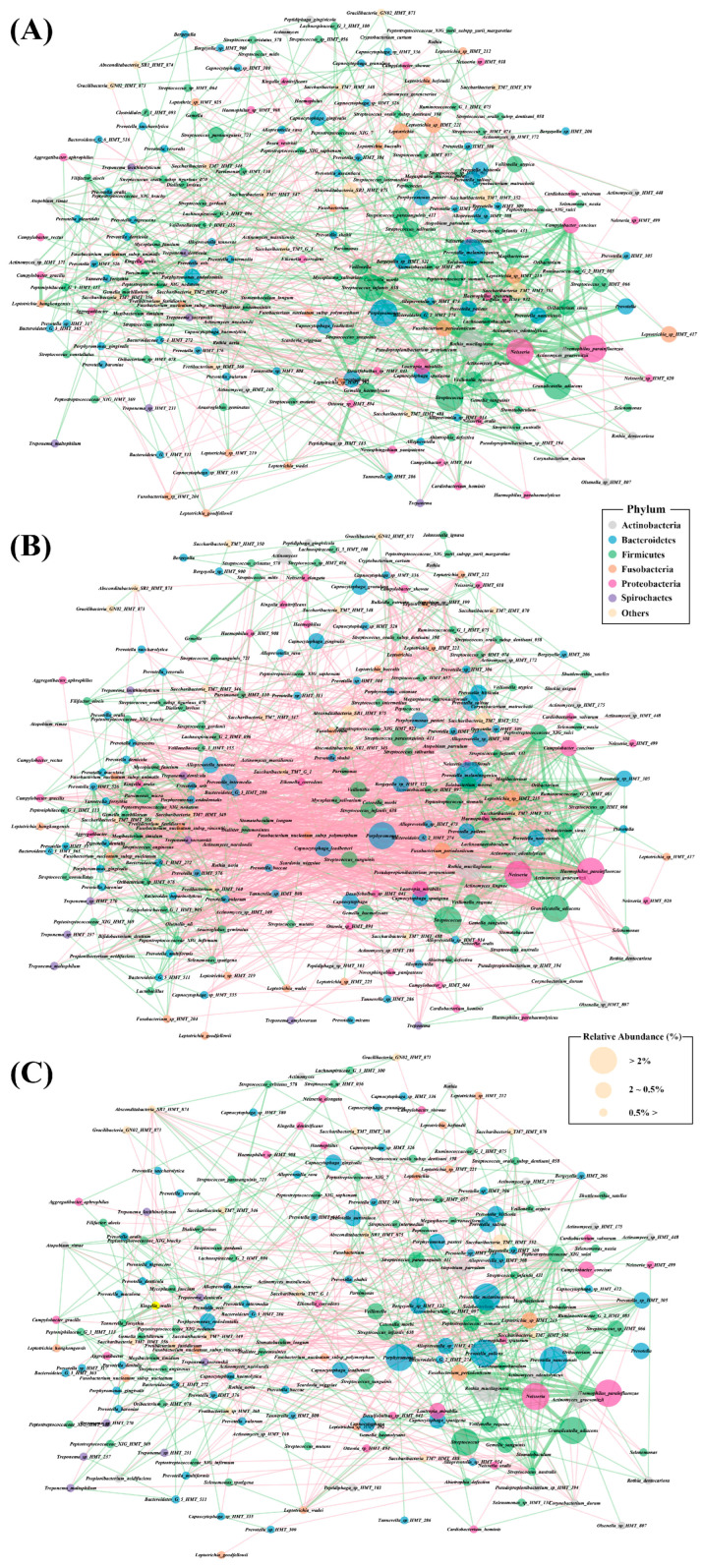
Salivary microbiome network analysis of (**A**) healthy, (**B**) erosive OLP, and (**C**) non-erosive OLP samples. Bubbles represent species and colors indicate phyla as described in the legend. A connection between two bubbles indicates a correlation between the two species. Positive correlations between species are indicated by green lines and negative correlations by red lines.

Microbial interaction networks were constructed using abundance profiles to identify potential interaction patterns. Network analysis revealed distinguishable bacterial interaction patterns among the three groups. The HC group exhibits a dense positive connection among commensal oral bacteria, including *Streptococcus*, *Neisseria*, *Haemophilus*, *Veillonella*, and *Rothia* species (Figure 5A). The NE-OLP group showed less dense interactions among commensal oral bacteria, as well as less dense negative correlations with pathogenic bacteria, including *Tannerella forsythia*, *Porphyromonas gingivalis*, *P. endodontalis*, *Filifactor alocis*, *Treponema socranskii*, and *T. denticola* (Figure 5C). Interestingly, E-OLP shows dense positive interactions among both commensal oral bacteria and pathogenic bacteria, as well as intense negative correlations between commensal oral bacteria and pathogenic bacteria (Figure 5B).

## 4. Discussion

This study aimed to investigate the microbial community in the saliva of patients with OLP, both with and without erosive lesions, and compare it to that of HC. In this study, the results showed that there was no difference in bacterial species diversity between OLP and HC, but there was a difference in composition (Figure 1). Consistent with previous studies, there were no statistically significant differences in bacterial alpha diversity between the three groups, as measured by the Chao1 and Shannon indices [26,27]. Beta diversity did not differ between the OLP groups, but it did differ between E-OLP and NE-OLP compared to HC. Yu et al. [28] showed a difference in beta diversity between E-OLPs and NE-OLPs, whereas Wang et al. [27] found no such difference, which is consistent with the results of this study. This discrepancy may be attributed to differences in primer selection in sequencing methods, bioinformatic analysis methods, and the number of subjects included in the studies. At the phylum level, the most commonly observed bacteria are Firmicutes, Bacteroidetes, Proteobacteria, Actinobacteria, and Fusobacteria. These findings are consistent with previous research [29,30,31]. While there was variation in composition among studies, in this study, the proportion of Bacteroidetes in the OLP group was higher than in the HC group (Figure 2A). This finding is in line with the results of Choi et al. [32], in which there was an increase in Bacteroides in patients with OLP compared to HC [32]. Bacteroidetes is a gram-negative bacteria considered a significant clinical pathogen, commonly observed in anaerobic infections. Bacteroidetes can induce anti-inflammatory T cell lymphocytes with their antigens in the intestinal tract. Additionally, they have been found to be abundant in conditions such as endocarditis, meningitis, septic arthritis, and osteomyelitis [33,34]. 

At the genus level, the proportion of *Prevotella* was higher compared to HC (Figure 2B). In general, the most abundant bacterium in HC is *Streptococcus*, but in the OLP group, the proportion of *Streptococcus* decreased and *Prevotella* was the most abundant [32]. *Prevotella* spp. are anaerobic gram-negative bacteria belonging to the Bacteroidetes phylum. They are commonly found as commensal colonizers at mucosal sites, as well as in saliva and several oral sites. *Prevotella* strains are generally considered commensal bacteria due to their extensive presence in the healthy human body and their rare involvement in infections. However, recent studies have linked an increased abundance of *Prevotella* and specific strains to inflammatory disorders. This suggests that some strains may exhibit pathobiontic properties. It is important to note that these findings are still emerging and require further investigation. Recent research has shown that the presence of *Prevotella* in biofilms of gingivitis and periodontitis can cause periodontitis, similar to *P. gingivalis*, which has traditionally been the focus [35]. In vitro studies have demonstrated that *Prevotella* has a greater ability to induce proinflammatory cytokines such as IL-6, IL-8, and tumor necrosis factor-α (TNF-α) compared to strict commensal oral bacteria and even *P. gingivalis* [36]. Components of bacterial cells activate Toll-like receptors (TLRs), with TLR4 primarily recognizing gram-negative bacteria [37]. Stimulation of TLR4 can lead to the production of proinflammatory cytokines, anti-inflammatory cytokines, and chemokines, as well as the initiation of inflammatory processes. Additionally, TLR signaling by *Prevotella* spp. can activate alternative NF-kB signaling [38].

In Salem et al.’s [39] study, the transcription of TLR4 was found to be upregulated in the oral epithelium of patients with OLP when compared to HC. This led to the reinforcement of TLR4 reactivity by the recruitment of T lymphocytes, resulting in a proinflammatory loop cycle. Additionally, OLP patients had higher concentrations of NF-κB-dependent cytokines TNF-α, IL-1α, IL-6, and IL-8 compared to HC [40,41,42]. Previous studies indicate that *Prevotella* may act as an antigen for T cell-mediated inflammation in OLP.

In the E-OLP group, *Prevotella denticola* and *Treponema socranskii*, which are also frequently observed in patients with chronic periodontitis [43], were among the significant taxa. Several studies have reported a correlation between OLP and periodontitis. Periodontitis is an inflammatory disease mediated by the host, which begins with the formation of bacterial biofilm and leads to gingival inflammation. OLP is also an inflammatory disease caused by an unknown antigen and is often painful, particularly in the case of E-OLP, which makes oral hygiene challenging. This difficulty leads to the accumulation of plaque and calculus, increasing the risk of long-term periodontal disease [44]. Conversely, the presence of these plaques and calculus can act as antigens and exacerbate OLP, and adequate plaque control has been shown to improve symptoms in patients with OLP with gingival involvement [45,46,47]. The relative proportions of *Treponema* spp. and *Porphyromonas* spp. were also higher in this study. This suggests that bacteria associated with periodontitis may also contribute to the inflammatory response in OLP (Figure 3).

Interestingly, *Capnocytophga* and *Peptococcus* were relatively more abundant in E-OLP but less abundant in NE-OLP compared to healthy subjects (Figure 4). *Capnocytophga* and *Peptococcus* are commonly observed in patients with gingivitis and halitosis, suggesting that NE-OLP may be less difficult to control than E-OLP and may be as well controlled in healthy individuals [48,49]. Thus, it may also be important to differentiate between E-OLP and NE-OLP when investigating, planning treatment, and managing patients with OLP.

This study has several limitations. Although the sample size is larger than in previous studies (with an average of 20 patients per group), a larger study is needed to account for other subject-related factors. Secondly, this study did not evaluate the periodontal status of OLP patients, and it was not possible to completely distinguish the periodontal disease from OLP. However, OLP patients before treatment are almost always accompanied by periodontitis due to pain, making it challenging to separate the two conditions. Thirdly, the direct causal role of these bacteria in the development and exacerbation of OLP has not been established. Nevertheless, we believe this study is valuable, as it contributes to the growing body of research at the intersection of OLP and microbiome studies, which have yet to yield consistent results due to variations in research methods.

## 5. Conclusions

In conclusion, the microbiome of the saliva in OLP patients differed from that of the HC group. Additionally, differences in the microbiome were identified between the E-OLP and NE-OLP groups. The increase in the proportion of certain bacterial species in the oral microbiome suggests that they may exacerbate the inflammatory response and act as antigens for OLP. In addition, our findings on the relative abundance of bacteria in OLP were different from previously reported studies, suggesting that further research on the salivary microbiome of OLP is needed.

## Figures and Tables

**Table 1:** Patient characteristics.

	HC (*n* = 30)	E-OLP (*n* = 25)	NE-OLP (*n* = 35)	*p*-Value
Age (mean ± SD)	55.13 ± 10.75	58.44 ± 10.17	55.66 ± 12.75	0.525
Male/female	3/27	9/16	9/26	0.069
NRS		4.66 ± 2.52	3.54 ± 2.09	0.067
No. of teeth (mean ± SD)	25.29 ± 4.45	23.48 ± 7.17	26.17 ± 4.53	0.170
REU score/No. of gingival erosive lesion		9.82 ± 5.18/12	2.34 ± 1.45/13	
Cigarette smoking	0	1	2	
Alcohol drinking	0	0	0	
Diabetes	1	3	2	

HC, healthy control; E-OLP, erosive oral lichen planus; NE-OLP, non-erosive oral lichen planus; NRS, numeric rating scale.

## Data Availability

The raw sequencing data have been deposited in NCBI GenBank under BioProject ID PRJEB73312.
